# Optimized design of a nanostructured SPCE-based multipurpose biosensing platform formed by ferrocene-tethered electrochemically-deposited cauliflower-shaped gold nanoparticles

**DOI:** 10.3762/bjnano.6.187

**Published:** 2015-09-01

**Authors:** Wicem Argoubi, Maroua Saadaoui, Sami Ben Aoun, Noureddine Raouafi

**Affiliations:** 1 University of Tunis El-Manar, Chemistry Department, Laboratory of Analytical Chemistry and Electrochemistry (LR99ES15), campus universitaire de Tunis El-Manar 2092, Tunis, Tunisia; 2 Department of Chemistry, Faculty of Science, Taibah University, PO. Box 30002 Al-Madinah Al-Munawarah, Saudi Arabia

**Keywords:** cauliflower-shaped gold nanoparticles, enzymatic detection, IgG sensing, nanotechnology, optimized design, screen-printed carbon electrode (SPCE) nanostructuration

## Abstract

The demand for on-site nanodevices is constantly increasing. The technology development for the design of such devices is highly regarded. In this work, we report the design of a disposable platform that is structured with cauliflower-shaped gold nanoparticles (cfAuNPs) and we show its applications in immunosensing and enzyme-based detection. The electrochemical reduction of Au(III) allows for the electrodeposition of highly dispersed cauliflower-shaped gold nanoparticles on the surface of screen-printed carbon electrodes (SPCEs). The nanostructures were functionalized using ferrocenylmethyl lipoic acid ester which allowed for the tethering of the ferrocene group to gold, which serves as an electrochemical transducer/mediator. The bioconjugation of the surface with anti-human IgG antibody (α-hIgG) or horseradish peroxidase (HRP) enzyme yields biosensors, which have been applied for the selective electrochemical detection of human IgG (hIgG) or H_2_O_2_ as model analytes, respectively. Parameters such as the number of sweeps, amount of charge generated from the oxidation of the electrodeposited gold, time of incubation and concentration of the ferrocene derivatives have been studied using cyclic voltammetry (CV), electrochemical impedance spectroscopy (EIS) and scanning electron microscopy (SEM). Selectivity and specificity tests have been also performed in the presence of potentially interfering substances to either hIgG or H_2_O_2_. Results showed that the devised immunosensor is endowed with good selectivity and specificity in the presence of several folds of competitive analytes. The enzyme-based platform showed a good catalytic activity towards H_2_O_2_ oxidation which predestined it to potential applications pertaining to enzymatic kinetics studies. The levels of hIgG in human serum and H_2_O_2_ in honey were successfully determined and served as assessment tools of the applicability of the platforms for real samples analysis.

## Introduction

Simplicity of design, cost-effectiveness and lightweight are among the most important requirements for the design of devices that can be used for the development of on-site analytical methods [1–3]. Rapid diagnostics in war zones, remote areas or on-field monitoring of warfare agents, explosives, pesticides and herbicides are few among many applications of such a technology. The elaboration of sensors to satisfy these needs is highly regarded [4–5]. Screen-printed electrodes on polymeric substrates using various types of conductive inks can be considered as one of the most promising routes for the development of cost-effective, disposable biosensors [4].

Electrochemical (bio)sensors are inherently endowed with several attracting features which are useful for various technological applications [6–8]. Enhancement of their detection capabilities can be easily achieved by modification with various types of nanomaterials to improve the electron-transfer rates between the redox center and the electrode and to procure catalytic effects which allow them in fine to reach the very low concentrations of, e.g., biologically active analytes and disease-related biomarkers [9–10]. Many electrochemical biosensors use ferrocene to transduce the biological reactions into readily measurable electrical signals [11–12]. For instance, Chen and Diao developed a glucose biosensor using gold nanoparticles that were tethered with a β-cyclodextrin/ferrocene inclusion complex as a transducing system. Connection between gold and the ferrocene host was achieved thanks to a thiol terminal group [13]. The ferrocene complex acted as an electron shuttle allowing for glucose oxidation at low potentials. Li et al. prepared a nanocomposite consisting of reduced graphene oxide hybridized with electrochemically co-reduced gold nanoparticles and ferrocene as a sensitive immunosensor of breast cancer biomarkers [14]. Very recently, Mars et al. showed that the aggregation of gold nanoparticles through a ferrocene-based bipodal ligand allowed for the preparation of a new kind of potential-shifting biosensors working differently from classical FET potentiometric sensors [15]. The system can be used to determine low levels of human immunoglobulin G (hIgG) over a large linear range. Graphene functionalized with ferrocene through a ethylenediamine spacer was prepared by Fan et al. and used as an efficient electron transfer (ET) shuttle to build a sensor for H_2_O_2_ [16]. Also, a nanocomposite formed with ferrocene-branched silica material reduced graphene oxide and glucose oxidase entrapped in chitosan matrix and was used for sensitive glucose determination [17].

**Figure 1:**
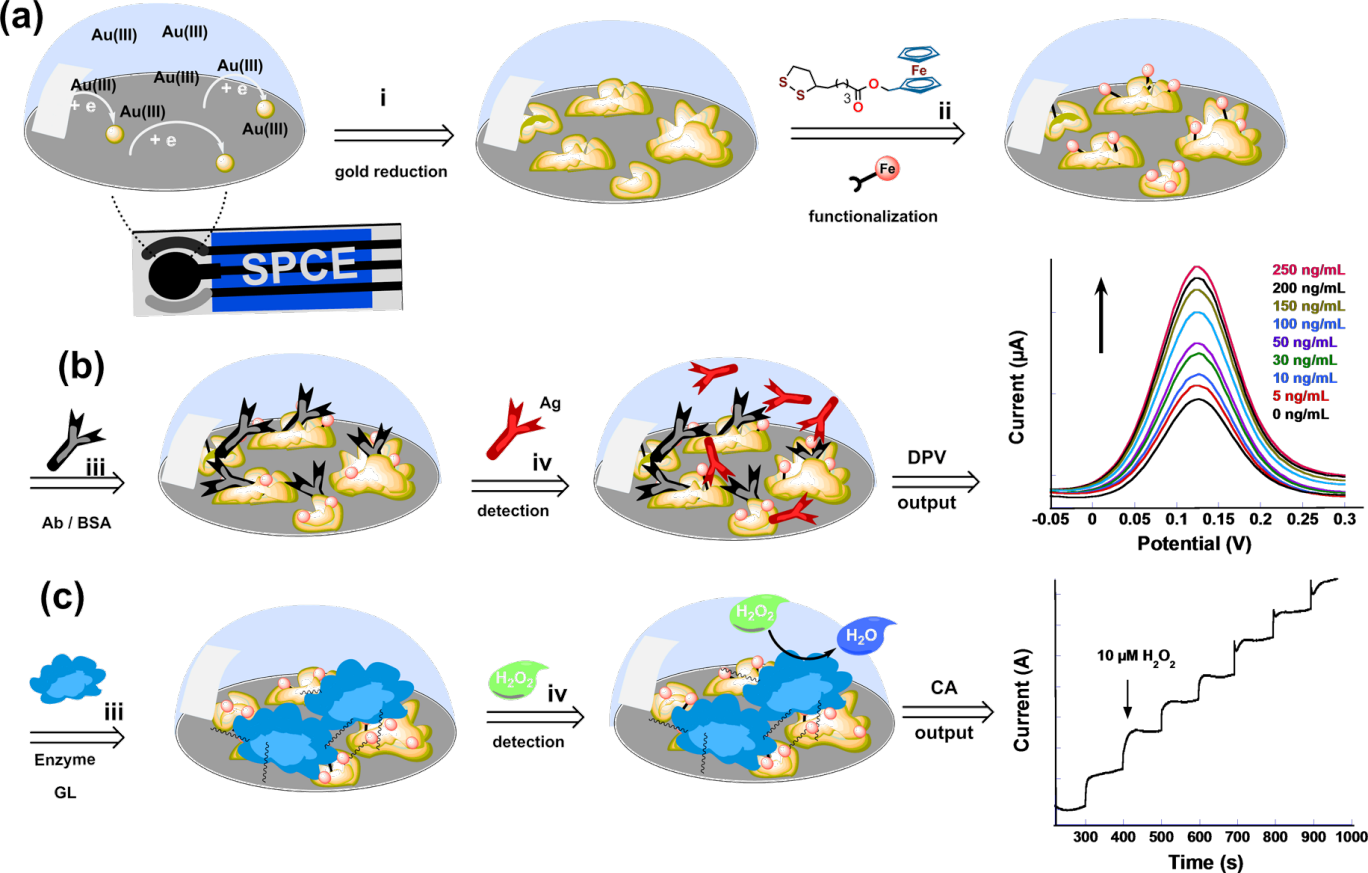
Schematic representation of: (a) the stepwise preparation of the cauliflower-shaped gold nanoparticles-modified SPCEs and their subsequent modification with the ferrocene derivatives, (b) the electrode modification with antibody and hIgG antigen detection by DPV and (c) the electrode modification with HRP enzyme and chronoamperometric detection of hydrogen peroxide.

In this paper, we report the optimization of the stepwise preparation of disposable SPCE-based biosensors using electrochemically deposited cauliflower-like gold nanoparticles (Figure 1). The optimized platform containing ferrocene-functionalized gold nanoparticles bioconjugated with anti-human IgG antibody or HRP enzyme was used for the detection of hIgG and H_2_O_2_, respectively. The large surface area of the nanostructured electrodes allowed one to detect low concentrations of the analytes. The selectivity and specificity were evaluated against the interference of competitive proteins using a panel of electrochemical techniques such as cyclic voltammetry (CV), differential pulse voltammetry (DPV) and electrochemical impedance spectroscopy (EIS). Application of the two platforms for the detection of hIgG and H_2_O_2_ respectively in human serum and in honey are presented.

## Results and Discussion

### Preparation of the nanostructured platform

**Figure 2:**
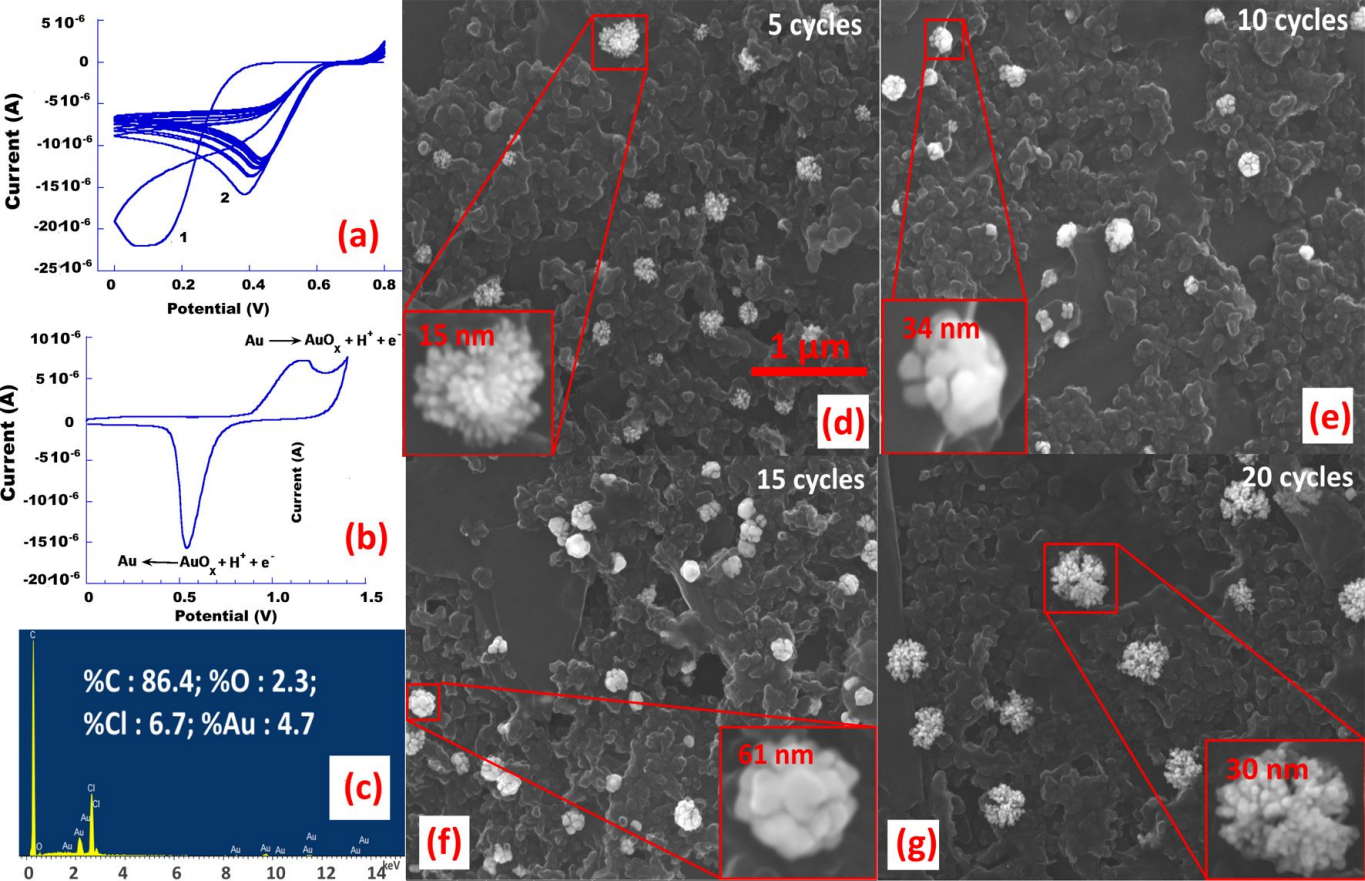
Nanostructuration and characterisation of the SPCE electrode, (a) cyclic voltamperometric deposition of gold nanoparticles from a 0.25 mM Au(III) solution (out of 15 cycles, only 8 cycles are shown for clarity); (b) Electrochemical oxidation and reduction of electrodeposited gold nanoparticles in a 0.5 M sulfuric acid solution and (c) EDX analysis of the nanostructured SPCE (5 cycles); SEM images of the modified carbon electrodes showing the presence of cauliflower-like gold nanoparticles obtained after: (d) 5 CV cycles, (e) 10 CV cycles, (f) 15 CV cycles and (g) 20 CV cycles. Inset: magnification of gold nanoclusters showing the sizes of the individual nanoparticles (scale 200 nm). Scale bar for Figure d–g: 500 nm.

To prepare the gold-nanostructured electrodes, we choose to deposit electrochemically the gold particles directly by reducing gold(III) on the surface of screen-printed carbon electrodes. This method has been very recently used to prepare fractal gold nanostructures for electrodes endowed with very large surface areas useful for the sensitive detection of apolipoprotein E, which is a protein biomarker for Alzheimer’s disease [18]. The preparation of the platforms was achieved in a straightforward manner in few steps. Firstly, home-prepared SPCEs were cleaned using a 0.5 M H_2_SO_4_ acid solution to remove the impurities adsorbed on the surface. Five cycles, where the potential was swept from 0.0 to 1.5 V, seemed to be enough to get sufficiently clean surface for gold electrodeposition. This step is crucial in order to get reproducible results. In a second step, the direct electrochemical reduction of a 0.25 mM solution of Au(III) cations to Au(0) on the carbon edges allowed for nanostructuring of the surface. Cyclic voltammetry was used to perform this step where the potential was swept from 0.8 to 0.0 V, since gold cations can be easily reduced in this potential window [19]. As it can be seen in Figure 2a, the first cathodic peak ascribed to the reduction of gold cations was observed at ca. 0.15 V (first cycle). In subsequent cycles (2nd to 15th) the potential shifted positively (from ca. 0.4 V to ca. 0.5 V) showing that it is much easier to reduce Au(III) on the nuclei of the formed gold nanoparticles than on the bare carbon substrate [19]. Moreover, using *I*–*t* transient current curves (Figure S1, Supporting Information File 1), it was proved that the mechanism of formation of gold nanoparticles is consistent with a nucleation/growth mechanism [20]. To the best of our knowledge, this is the first case where the nanostructuration is systemically studied in order to optimize the electrodeposition procedure. The gold electrodeposition on SPCE allowed us to obtain a modified electrode with an active surface as large as that of a bulk gold electrode with diameter of 1 mm. The surface calculation was done by assuming the formation of a gold oxide monolayer which is electrochemically reduced by consuming 386 µC/cm^2^ [21–23]. Although using a reduced amount of gold salt, an appreciable surface is obtained at a reduced cost to accommodate a large number of ferrocene derivatives and proteins for the purpose of developing disposable biosensors.

From Figure 2d, we can conclude that the nanoparticles preferrably grow on the edges of the carbon platelets because they contain hydroxyl and carboxyl reactive sites formed during the surface cleaning step [24]. After the electrodeposition, the electrode surface was cleaned with ultrapure water. Cyclic voltammetry performed in a 0.5 M sulfuric acid solution clearly shows two waves, the first one is related to gold oxidation occurring around ca. 1.2 V and the second one is related to its reduction peak appearing at ca. 0.6 V [21–23], thus providing proofs of the formation of gold nanoparticles onto the SPCE (cf. Figure 2b). This is further supported by SEM images (Figure 2d–g) and EDX analysis (Figure 2c) confirming that the nanostructuration provokes the formation of small gold nanoparticles which agglomerate into highly dispersed, more complex, cauliflower-shaped gold nanoparticles with the increase of the number of cycles. After 15 cycles, the particulates reach an average diameter of ca. 61 nm.

For electrodes modified by 15 sweeping cycles, EDX showed the presence of ca. 4.7% of metallic gold on the surface of the SPCE in addition to ca. 2.3% of oxygen which most probably originated from the terminal carbon oxygenated functions and gold oxides formed by the oxidation of freshly formed nanoparticles. The presence of chlorine (ca. 6.7%) can be explained by its entrapment between the carbon layers of the working electrode during the voltamperometric process.

### Effect of cyclic sweeps on the nanostructured surface

We further studied the effect of the number of cycles on the final nanostructured surface. For a reduced number of cycles, we observed that the number and size of gold nanoparticles were relatively small (Figure 2d and Table S1, Supporting Information File 1). When the number of cycles increased, the density of particles (i.e. the number of nanoparticles per square micrometer) raises rapidly (Figure 2e and Figure 2f) up to 15 cycles, then dramatically decreases for higher number of cycling sweeps because the neighbouring nanoparticles fused into bigger cauliflower-shaped nanostructures as can be observed in Figure 2g. As estimated from the counting of the particles on several SEM micrographs, the approximate size of individual gold nanoparticles increased rapidly from ca. 15 to ca. 61 nm for 5 to 15 cycles then dropped to ca. 30 nm thereafter. Probably, the adjacent small gold nanoparticles fused into bigger ones and served as nucleation sites for larger particles. From Table S1 (Supporting Information File 1), the mean number of particles as well as their density is almost the same for 5 and 10 cycles and both increased by almost 50% after 15 cycles. For more cyclic sweeps, the number and the density of deposited nanoparticles dropped noticeably by 50%, which confirms the fusing of adjacent particles. Thus, we conclude the drastic effect of the cyclic sweeps on the obtained nanostructures which should be carefully optimized before stepping further.

### Electrochemical properties of the nanostructured surface

To further investigate the properties of the modified carbon surface, it was characterized using cyclic voltammetry, chronocoulometry and electrochemical impedance spectroscopy.

### Cyclic voltammetric and chronocoulometric studies

**Figure 3:**
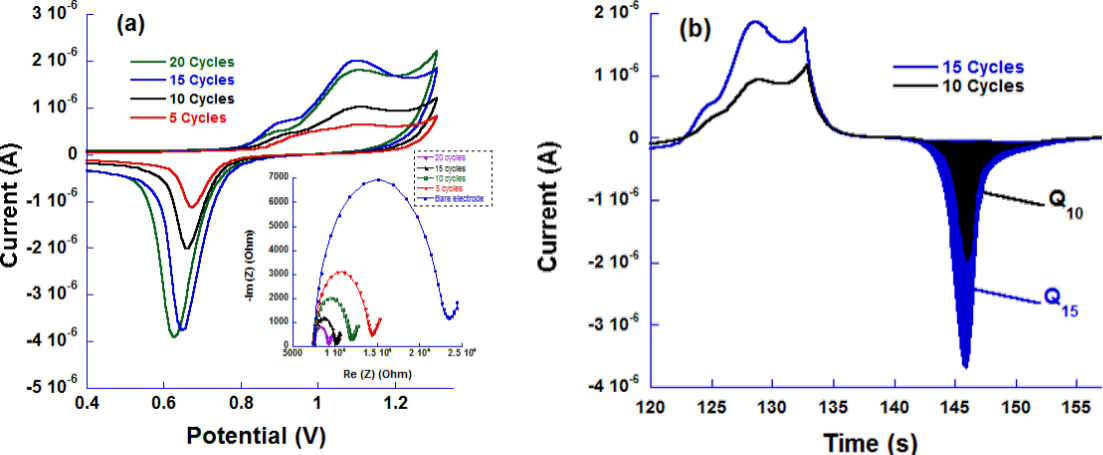
(a) CV curves of four different electrodes nanostructured for 5, 10, 15 and 20 cycles along with the respective Nyquist plots (inset) and (b) *I*–*t* curves after baseline correction showing the quantity of charge (*Q*) determination for 10 and 15 cyclic sweeps.

CV studies showed that the currents generated from gold redox activity are also dependent on the number of cycles as it is depicted in Figure 3a. One can observe both anodic and cathodic currents increase with increasing number of electrodeposition scans. The reduction peak can be used to characterize the quality of gold surface and to measure the active surface area of gold electrodes. During the ongoing scan, gold is oxidized in sulfuric acid to form Au(III) cations and gold oxide (AuO*_x_*) species which are reduced to Au(0) during the backward scan [25]. The amount of charge consumed during the reduction of the oxidized gold can be obtained from the surface integration of the current versus time curves (*I*–*t* curves) derived from the cyclic voltammograms which is a useful tool to estimate the effective sensing area of the as-prepared nanostructured electrodes (Figure 3b) [26].

The amount of charge for different electrodes are summarized in Table 1. We can observe that the amount of charge increases with the increasing number of cycles due to the accumulation of gold on the electrode surface. For instance, the charge quantity nearly doubled from 5 to 10 cycles and same from 10 to 15 cycles (filled areas in Figure 3b). After 20 cycles, it was about four times higher than that obtained for 5 cycles. Although the variation in the density of the as-deposited cauliflower-like gold nanoparticles was not monotonic, we can conclude the observed trend of the quantity of charge is a clear indication that the overall amount of deposited gold increases with increasing number of deposition cycles giving larger active surface areas useful to tether a high number of ferrocene moieties for more sensitive devices.

**Table 1:** Faradaic amount of charge (*Q*) resulting from gold reduction, percent variation of the amount of charge, charge transfer resistance (*R*_CT_) of the surface and percent variation of *R*_CT_ as function of the number of electrodeposition cycles.

number of cycles	5	10	15	20

quantity of charge calculated for the gold reduction / µC	2.463	4.669	9.111	9.892
% of *Q* variation	0	89.6^a^	269.9^a^	301.6^b^
*R*_CT_ / Ω	6915	3565	2873	2611
% of *R*_CT_ variation^c^	0	48.5	58.4	62.2

^a^Calculated as 100·(*Q_j_* − *Q_i_*)/*Q_i_*, *j* = *i* + 5, *i* = number of cycles; ^b^calculated in relation to 5 cycles; ^c^calculated as 100·(*R*^0^_CT_ − *R^i^*_CT_)/*R*^0^_CT_, R^0^_CT_ is for 5 cycles and R^i^_CT_ for 10, 15 and 20 cycles.

A number of cyclic sweeps equal to 15 is ideal to obtain a nanostructured surface with high density of well-dispersed nanoparticles and a surface of about 7.8 × 10^−3^ cm^2^ corresponding to a 1 mm diameter bulk gold electrode.

### Study by electrochemical impedance spectroscopy

Gold is a more conductive material than carbon, so the voltamperometric deposition of gold onto the carbon surface is expected to drastically alter its conductivity, which could be easily monitored using faradaic EIS measurements. This is clearly depicted in the Nyquist plots (inset in Figure 3a) of the different electrodes, recorded in the presence of a 5 mM [Fe(CN)_6_]^4–^/[Fe(CN)_6_]^3−^ solution used to probe the surface conductivity, where the *R*_CT_ decreases dramatically upon only a five-cycle deposition and continues to decrease monotonously with an increasing number of performed cycles as a consequence of increasing densities of the deposited gold particles. The outcome of the electrochemical impedance spectroscopy thus corroborates the coulometric studies results (Table 1).

In the present study, a number of 15 cycles was chosen for subsequent investigations since it gave the highest density of cauliflower-like gold nanoparticles with the best surface conductivity. These two parameters are eventually a prelude to larger active surface areas which would allow the tethering of a greater number of ferrocene moieties.

### Immunosensing of human IgG

The ferrocene derivative (FcD) was obtained by Steglich DMAP-catalyzed esterification between lipoic acid and ferrocene methanol using DCC as a condensing agent according to previously published reports [15,27].

### Immunosensor preparation

It is well known that gold has a high affinity toward sulfur-terminated compounds [28]. Several experiments showed that gold nanostructured electrodes should be incubated for at least 6 h in a 1 mM ferrocene ester solution to get the highest currents of the ferrocene oxidation peaks (Figure S2, Supporting Information File 1). Electrochemically assisted method of lipoic acid deposition was also tested [29] to improve the modification and shorten the incubation time. However, the obtained results from the passive method were better.

Cyclic voltammetry showed a reversible CV profile related to the oxidation of the ferrocene moiety to the ferrocenium cation at ca. 0.14 V and a reduction peak at ca. 0.09 V. The peak-to-peak separation of ca. 50 mV and the *I*_pc_-to-*I*_pa_ current ratio close to unity suggest that ferrocene keeps its reversibility even when adsorbed onto the gold surface. The slope of the plot representing the logarithm of current versus the logarithm of the scan rate was found to be ca. 0.94 which is close to unity, suggesting an adsorption-controlled process [30], which is consistent with a self-assembled monolayer of alkyl groups bearing terminal electroactive redox centers.

**Figure 4:**
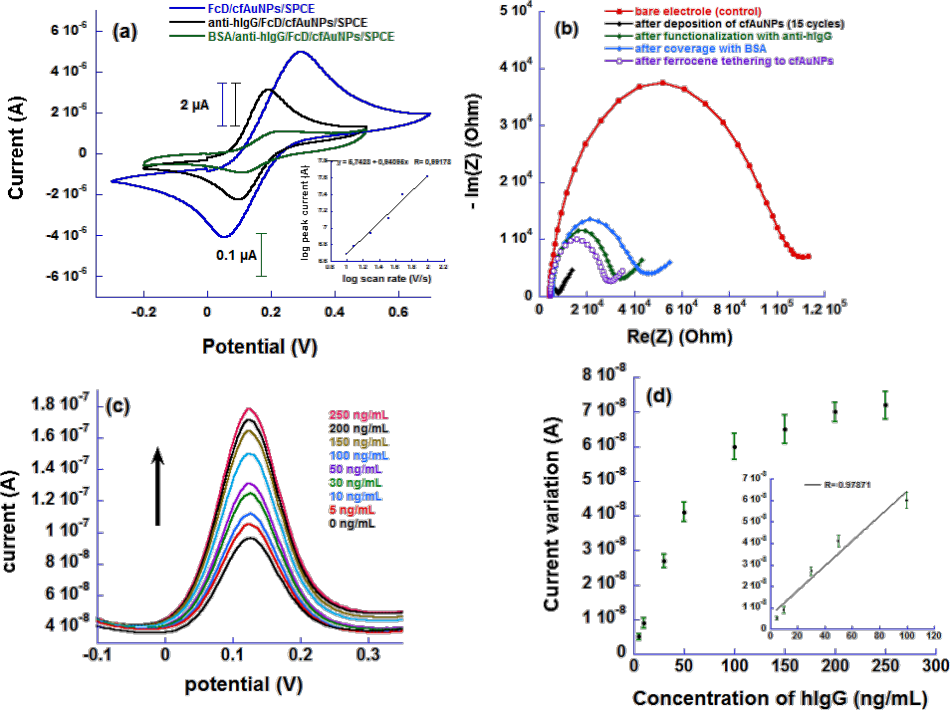
(a) CV curves of functionalization of cfAuNPs by FcD (blue), conjugation with the Ab (black), coverage with BSA protein (green). The inset shows the the logarithm of the peak current as a function of the logarithm of the scan rate, (b) EIS characterization of the stepwise surface modification, (c) Variation of DPV current response upon the antigen addition and (d) Calibration curve for hIgG biosensing (the inset shows the linear concentration range).

The FcD/cfAuNPs/SPCE were conjugated with the antibody then covered by 3% BSA to avoid the oxidation of bare cfAuNPs (Figure 4a). The CV curves of FcD prior and after addition of the antibody and BSA have similar shapes with a noticeable decrease in current densities showing that the formation of a protein layer hindered the ET process. Faradic EIS showed that the surface modification by gold induces a dramatic decrease of *R*_CT_, which slowly increases after the stepwise modification by ferrocene derivative, the antibody and BSA (Figure 4b). In the first step, electrodeposition of gold nanoparticles on the carbon surface provokes a sharp decrease of the surface resistivity because of the higher conductivity of gold. The subsequent self-assembly of the ferrocene derivative induces a roughly 4-fold increase of the surface resistivity because of the total passivation of the gold surface by a sulfur layer. Further modification with the antibody and BSA increases the surface impedance due to the electron transfer from the redox probe present in the solution. Thus, the ferrocene covered by a protein layer can be expected to transduce the recognition event between the antibody and the corresponding antigen (Ag).

As it was described above, this method can be used for the straightforward preparation of bioelectrodes. In this case, the direct deposition of gold nanostructures allowed us to avoid using pre-formed gold nanoparticles and to develop electrodes with a large surface area by forming cauliflower-shaped nanoparticles, which can accommodate up to ca. 1.83 × 10^12^ ferrocene molecules. The large number of ferrocene units is expected to result in relatively large currents for a sensitive determination of the analytes and permits to use them for electronic mediation between the enzyme redox center and the electrode surface.

### Determination of hIgG levels

DPV studies showed that successive additions of hIgG aliquots containing known amounts of the antigen to the immunosensor induce current increases of the ferrocene oxidation peak which could attributed to the sensor immunoresponse as a result of an Ag–Ab immunocomplex formation (Figure 4c). The DPV current increase continued up to 250 ng/mL of hIgG denoting a wide dynamic range and the calibration curve is given in Figure 4d (the inset shows the linear range curve). We can speculate that the current increase is due to a larger protein layer that induces an increase of the electron-transfer rate from the ferrocene to the electrode surface through the highly conductive gold nanostructures. The antigen forms a third layer over the deposited gold which probably does not impede the electron transfer since it occurs at the inner ferrocene layer directly linked to the gold. Because during the detection step the number of ferrocene units remains unchanged, the number of electron transferred to the electrode should be the same. Therefore, we can reasonably think that the current increase is due to higher electron-transfer rates resulting from a conformational change of the flexible carbon chain bearing the ferrocene which gets closer to the electrode surface after adding the antigen (i.e., hopping electron-transfer mechanism). Several recent works support the fact the electron-transfer rates can be enhanced by the conformational changes especially of long alkyl chains and biomolecules used to tether ferrocene to a gold surface [31–34].

The current variation stopped at high concentration because of the saturation of all the recognition sites of the immunosensor. The plot of potential variation vs the amount of Ag gives a straight line (R^2^ = 0.97871) (Figure 4d) which confirmed the reproducibility of the measurements [35]. The limit of detection (LoD) and the limit of quantification (LoQ) were calculated using the formulae LoD = (3 × *s*_b_)/*S* and LoQ = (10 × *s*_b_)/*S*, respectively, where *s*_b_ represents the standard deviation of the blank signal and *S* represents the sensitivity of the calibration curve. The latter is determined from the slope of the plot of the linear range between 0 and 50 ng/mL (i.e., ca. 0.82 nA·mL/ng). These limits were estimated to be ca. 0.11 ng/mL and ca. 0.39 ng/mL, respectively.

The results presented here are in a sharp contrast with those already reported recently by our group [15]. In both cases, ferrocene-modified gold nanoparticles were used. In this particular case, the particles are further tethered to the electrode surface and we observed an amperometric response (current variation) while, when the ferrocene-modified particles were used in colloidal solution, we observed an anodic shift of the ferrocene peak potential which is ascribed to the change in the electrical charge near the ferrocene groups after the addition of antigen. One can conclude that ferrocene is useful as a transducing system to follow biosensing events but foreseeing the exact electrochemical behavior (amperometric vs potentiometric) remains very difficult.

Furthermore, the redox center directly linked to the gold surface, through the formation of a ferrocene-terminated self-assembled monolayer, results in good ET rates from ferrocene to the electrode surface. This property allows using the modified surface to act as a transducer for immunosensing event. More importantly, the ferrocene, being attached to the surface through two gold–sulfur bonds, greatly limits ferrocene leakage into solution and keeps the sensors active. Higher surface coverage allows higher loading in ferrocene and thus widens the dynamic ranges.

### Selectivity and specificity of the immune response

**Figure 5:**
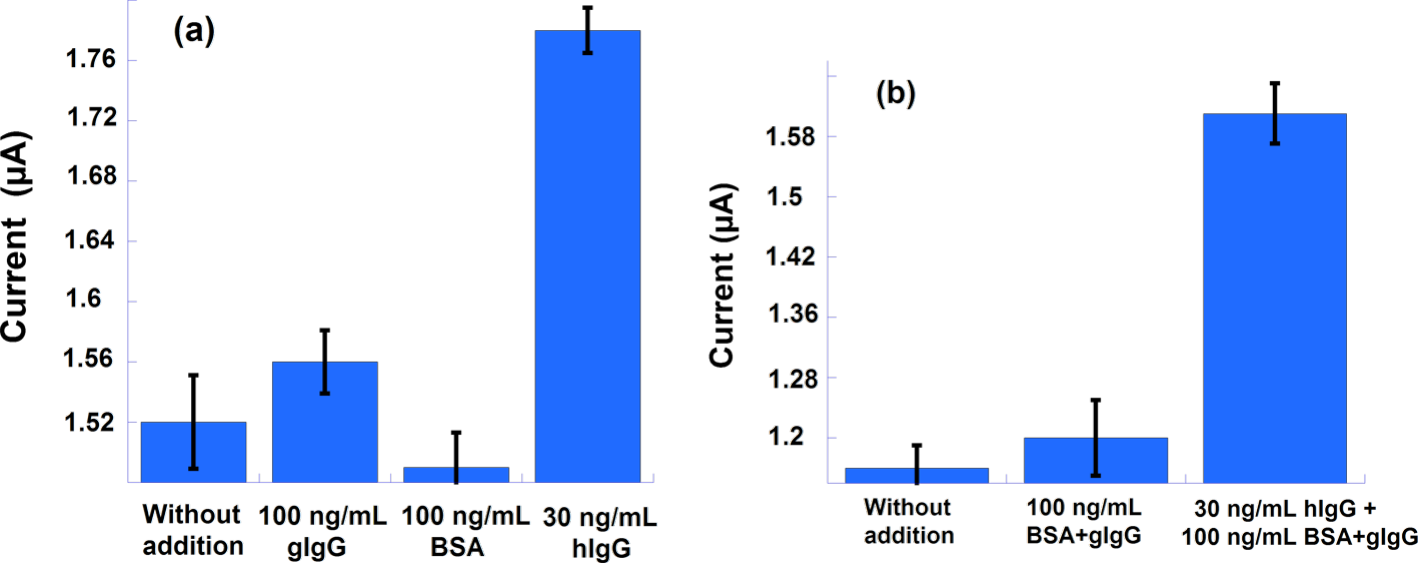
(a) Selectivity tests: current responses induced by the addition of gIgG, BSA or hIgG protein and (b) specificity studies: current variation related to the addition of gIgG/BSA and hIgG/gIgG/BSA mixtures showing a large current increase due to the presence of hIgG.

Selectivity and specificity are among the prominent advantages of immunosensors. Several experiments have been undertaken to demonstrate that the observed current response is only ascribed to the immunoresponse. Selectivity and specificity were measured against the interference of BSA and gIgG as competing proteins to the hIgG analyte. The addition of 30 ng/mL of hIgG and 100 ng/mL from the other proteins had little effect on the oxidation current of ferrocene, which remained almost unchanged compared to the current observed with the same amount of hIgG. In addition, to illustrate the immunoresponse specificity, the three proteins were added at once and the resulting DPV current was compared to that obtained from the hIgG alone. The overall results are summarized in the histogram given in Figure 5a (DPV curves are given in Figure S3, Supporting Information File 1). The small variation in the ferrocene oxidation current could be due the structural similarity between the hIgG and the corresponding antibody gIgG used as an interfering protein. The histogram displayed in Figure 5b shows the results from the specificity tests, it can be seen that the addition of a mixture of proteins provokes a strong current increase while in the absence of the specific analyte the current variation is weak denoting a very good specificity of the device. The current rise in the latter case is probably due to structural similarity between gIgG (i.e., antibody to hIgG antigen produced in goat) and the other immune proteins.

### Enzyme-based biosensing

The oxidoreductase family of enzymes is an important class of enzymes that catalyze redox reactions. They are well-adapted for the construction of electrochemical enzyme-based biosensors for many applications such as blood sugar level measurement, phenol oxidation, and the detection of pesticides and herbicides [36–40]. The second generation enzyme-based biosensors rely on mediators to shuttle electrons from the inner redox moiety of the enzyme to the electrode surface or vice versa. Ferrocene proved to be a good electron mediation agent for such use [13].

### Preparation of the enzyme-based sensor

**Figure 6:**
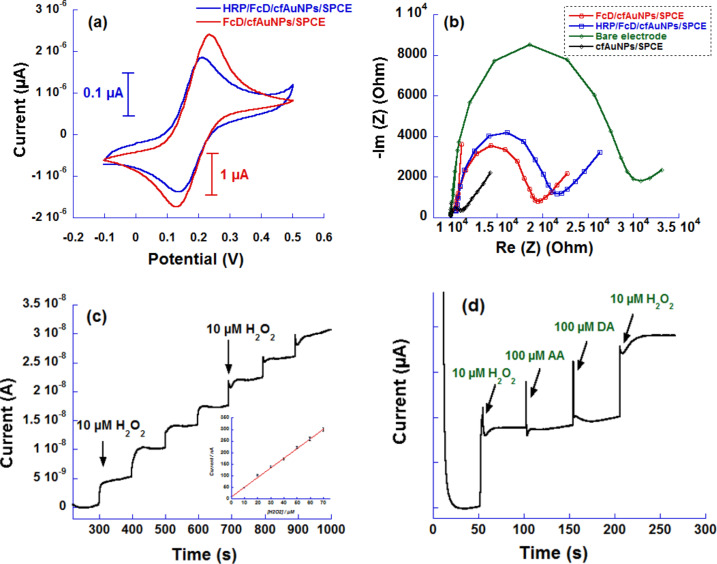
(a) CV curves of functionalized cfAuNPs by the FcD (red) and its conjugation to the HRP enzyme (blue), (b) EIS characterization of the stepwise surface modification, (c) amperometric response of the biosensor to H_2_O_2_ concentrations in a phosphate buffer solution of pH 7.4 at an applied potential of 150 mV (ferrocene reduction potential) upon successive additions of 10 µM of H_2_O_2_ at time intervals of 100 s (inset: experimental data and linear fitting curve) and (d) selectivity study of the enzymatic response in a PBS solution at an applied potential of 290 mV upon successive addition of 10 µM of H_2_O_2_ with 100 µM of dopamine (DA) and ascorbic acid (AA).

The biosensor was prepared by simply drop-casting a mixture of the HRP enzyme solution and chitosan as displayed in Figure 1. The chitosan was used as a matrix to retain the enzyme on the nanostructured surface [39]. The stepwise preparation was also characterized using CV and EIS spectroscopy. CV showed an effect similar to the addition of antibody where the current drops by tenfold after the enzyme casting. *R*_CT_ also increased after the enzyme casting, most probably due to the formation of an inhibiting layer of proteins as it was observed for the sequential modification during the preparation of the first biosensor (Figure 6a and Figure 6b).

### Selective detection of H_2_O_2_

The simplicity of design and the ease of layer-by-layer modification make this surface an excellent electrochemical sensing platform for hydrogen peroxide detection. Figure 6c shows typical chronoamperometric responses of the ferrocene moiety to the successive additions of H_2_O_2_ at an applied potential of +0.15 V. The inset in Figure 6c shows the linearity of the calibration curve along the different additions of H_2_O_2_ (R^2^ = 0.9989) with a sensitivity of ca. 4.13 nA/µM. The biosensor has a fast response time since the current rapidly reaches a steady state (about 10 s). At this potential, the ferrocene is maintained permanently at a reduced state (Fc), meanwhile the enzyme transfers electrons from the ferrocene to the analyte to reduce it. The formed ferrocenium cation (Fc^+^) is immediately reduced to its original form since the applied potential corresponds to the ferrocenium/ferrocene reduction potential [41–42]. The novelty in this bioelectrode concept is that the devised system is using a relatively large number of ferrocene units (ca. 1.83 × 10^12^ molecules) tethered to the highly conductive gold material, which results in fast electron-transfer rates from the redox moiety to the electrode surface. Furthermore, the ferrocene vicinity to the HRP enzyme is useful to constantly re-oxidize the enzyme FAD cofactor thus allowing the design of fast response biosensing devices endowed with a large dynamic range.

The LoD and the LoQ were calculated using the same criteria as described earlier for a sensitivity equal to 4.13 nA/µM and a linear range between 0 and 70 µM. The limits were found to be ca. 0.66 µM and ca. 2.20 µM, respectively, denoting that the device will be useful to determine sensitively low amounts of hydrogen peroxide at low working potentials comparatively to the direct reduction of H_2_O_2_ (ca. 1.50 V/SCE).

Moreover, the biosensor selectivity was examined using up to the tenfold amount of ascorbic acid and dopamine as interfering agents. From Figure 6d, one can see that the presence of these redox compounds did not significantly alter the amperometric response of the sensor and that is mainly attributed to the low working potential of +0.15 V.

### Comparison with literature

Table 2 presents the performance results gathered for various immuno- and enzyme-based sensors. Depending on the analyte, hIgG is usually sought to be detected in picogramm (ng/mL) levels and hydrogen peroxide in micromolar levels. From the table, we can see that the developed biosensors are more sensitive than most of those recently published in literature using ferrocene or quinone as electron mediators or immunosensing event transducers. The higher sensitivity can be probably ascribed to ferrocene directly attached to the electrode surface. The relatively limited dynamic range is probably due the low number of ferrocene molecules self-assembled to gold nanoparticles (about 0.4 nmole) limiting the turnover frequency during the hydrogen peroxide reduction.

**Table 2:** Structures and performance values of various biosensors reported in literature for the detection of hIgG and H_2_O_2_.

modified electrodes	analyte	redox mediator	LoD	dynamic range	reference

detection of hIgG					

AuNPs	hIgG	dopamine	0.25 ng/mL	0.82–90 ng/mL	[43]
IrO*_x_*^a^		hydroquinone	8.0	10–200	[44]
AuNPs/SPCE		ferrocene	9.4	50–200	[15]
AuNPs/SPCE		4-*t*-butylcatechol	3.1	11–205	[45]
Au colloid		[Fe(CN)_6_]^4−^/[Fe(CN)_6_]^3−^	4100	(15–328) × 10^3^	[46]
cfAuNPs/SPCE		ferrocene	0.11	0.11–50	this work

detection of H_2_O_2_					

montmorillonite colloid	H_2_O_2_	methylene green	0.4 μM	2–3000 μM	[47]
colloidal Au/ITO		—	8.0	20–8000	[48]
Au colloid		catechol	0.15	0.4–330	[49]
Fc/CPE^b^		ferrocene	0.1	0.1–10	[50]
Fc/PAPO/CPE^c^		ferrocene	—	0.6–20	[51]
cfAuNPs/SPCE		ferrocene	0.66	0.7–70	this work

^a^IrOx: iridium oxide; ^b^CPE: carbon paste electrode; ^c^PAPO: poly(3-aminophenol).

### Real sample analysis

The two devices have been successfully applied to the detection of hIgG and hydrogen peroxide in human blood serum and local honey [52] chosen as real complex samples, respectively. Before the analysis, the two samples of honey and serum were diluted to 10× and to 1000× in order to meet the dynamic ranges of their respective biosensors. The concentrations were measured and the obtained data are summarized in Table 3. Known amounts of hIgG and H_2_O_2_ were then added to the solutions and their concentrations were again measured (Figure S4, Supporting Information File 1). The calculated recovery percentages were found to be in the range of 105.4–106.5% and 102.0–103.0% with standard derivations of less than 4.3% and 3.8% for human IgG and hydrogen peroxide, respectively [53]. These high recovery values indicate good performances of the HRP/FcD/cfAuNPs/SPCE and the anti-hIgG/FcD/cfAuNPs/SPCE even for real samples containing high levels of interfering species.

**Table 3:** Results of the detection of hIgG and H_2_O_2_ in real samples of human blood serum and honey and recovery percentages after successive additions of both analytes.

samples	detected (P)	added (Q)	detected after addition (R)	% recovery 100 × (R − P)/Q (std deviation)

detection of hIgG				

serum	8.0	10	18.54	105.4 ± 3.5
	—	20	29.3	106.5 ± 4.3

detection of H_2_O_2_				

honey	12.9	10	23.1	102.0 ± 3.6
	—	20	33.5	103.0 ± 3.8

## Conclusion

In this work, we showed that the design of a disposable platform structured with cauliflower-like gold NPs (cfAuNPs) can be achieved in a stepwise manner and we tried to find the optimal conditions for its design. In a second step, we successfully applied it for the fabrication of two families of biosensors. The first one is devised for the hIgG immunosensing as a model analyte and the second one for the catalytic detection of H_2_O_2_. Both biosensors rely on ferrocene tethered to the gold nanoparticles to either transduce the recognition event or to catalyze the hydrogen peroxide reduction. The biosensors are endowed with good dynamic ranges, high sensitivities and were found to be selective and specific to the examined analytes. The biosensors were assessed for accurate determination of the analytes in complex matrix formed by human serum and honey. The platform is a prelude to a more specific application in the design of point-of-care and portable devices for use in remote areas and on-field monitoring of several types of analytes such as explosives and other warfare agents.

## Experimental

### Materials and apparatuses

α-Lipoic acid (≥98%), 1-ferrocenylmethanol (97%), dimethylaminopyridine (DMAP) (99%), HAuCl_4_^.^3H_2_O (99.9%), dicyclohexylcarbodiimide (DCC) (99%), sodium nitrate (≥98%), human IgG (hIgG, ≥95%), polyclonal anti-human IgG antibody (α-hIgG), polyclonal anti-human IgG antibody (gIgG, produced in goat), bovine serum albumin (98%), ascorbic acid, dopamine, H_2_O_2_, silica gel 60 mesh and F_254_ nm fluorescent silica-coated aluminum TLC plates were purchased from Sigma-Aldrich (Germany) and used as received without further purification. All solvents used were of analytical grade and were used without further purification. Human blood serum obtained from the blood of healthy persons and honey from the local market were used as real samples for the detection of hIgG and H_2_O_2_, respectively.

All voltammetric experiments (CV and DPV) and EIS measurements were recorded using a Metrohm Autolab PGSTAT M204 electrochemical workstation equipped with an FRA impedance module. Experiments were designed and data collected using Nova^®^ software. Custom-made screen-printed carbon electrodes (SPCEs) printed on polyethylene terephthalate (PET) sheets using a DEK-248 screen printer (DEK International) were employed to perform the electrochemical experiments. Each SPCE comprises of a 3 mm disk used as a carbon working electrode, a printed Ag/AgCl reference electrode and a carbon counter-electrode.

SEM micrographs and energy-dispersive X-ray spectroscopy (EDX) analysis were recorded using a FEI Quanta 650 FEG ESEM scanning electron microscope. NMR spectra were recorded in Bruker Advance 300 apparatus in CDCl_3_ at 300 MHz frequency. Chemical shifts are given in ppm using tetramethylsilane (TMS) as an internal reference. Deionized water, produced using Milli-Q system (>18.2 MΩ/cm) purchased from Millipore Inc., was used for the preparation of all solutions.

### Electrochemical measurements

Voltammetric measurements were carried out in triplicate by casting 25 µL of hIgG dissolved in a PBS solution (pH 7.4) on the modified SPCE working electrode. For all the immunosensing experiments, the CV and DPV curves were measured at a scan rate of 50 mV/s in the potential range between −0.1 and 0.4 V prior and after successive additions of 2 µL from stock solutions to achieve 5, 10, 30, 50, 100, 150, 200 or 250 ng/mL of hIgG on the working electrode surface. For the selectivity tests, 25 µL (i.e., 100 ng) of BSA or α-hIgG were cast onto the surface of the biosensor, the DPV current was measured and compared to that obtained for 30 ng of hIgG. Specificity was obtained from comparative measurements of currents obtained for 25 µL containing 100 ng of BSA and 30 ng of hIgG and 25 µL containing a mixture of the same amount of BSA, α-hIgG and gIgG.

For EIS measurements, after each step of the SPCE modification, 25 µL of a PBS solution containing 5 mM of [Fe(CN)_6_]^4−^/[Fe(CN)_6_]^3–^ were dropped on the electrode surface and the frequency was swept from 100 kHz to 0.1 mHz at an applied potential of 200 mV with an amplitude modulation of ±20 mV.

### Preparation of the biosensing platform

The ferrocene derivative (FcD) was prepared according to literature [15,27]. A 0.25 mM Au(III) solution in 0.1 M KNO_3_ was prepared by dissolving 100 µL of 0.01 M HAuCl_4_ and 4.9 mg of KNO_3_ in 300 µL of deionized water. The nanostructuration was performed by cycling the potential from 0.8 to 0.0 V at 50 mV/s sweep rate in order to reduce Au(III) to Au(0). Separately, 50 µL of a 0.1 M ethanolic solution of freshly synthesized FcD was added to 450 µL of a PBS solution to prepare a 10 mM FcD solution. The nanostructured electrode was incubated in the as-prepared solution for 6 h to allow the ferrocene tethering to the electrode surface. Finally, to prepare the immunosensor, 5 µL of a 10 µg/mL α-hIgG antibody solution was cast on the modified surface and allowed to dry at RT for 24 h. The non-adsorbed antibodies were washed away with PBS then ultrapure water and the unmodified gold was blocked by the addition of 5 µL of a 3% BSA solution.

The enzyme-based platform was prepared by drop-casting 5 µL of HRP solution (10 mg/mL) onto the ferrocene-modified cfAuNPs-structured electrode.

Determination of the concentrations of hIgG and H_2_O_2_ in real samples were performed by DPV and chronoamperometry using the anti-hIgG/FcD/cfAuNPs/SPCE and HRP/FcD/cfAuNPs/SPCE. To prepare the solutions, 1 and 100 µL of serum and honey were respectively dissolved in 1 mL of PBS solution (30 min sonication is necessary to obtain a homogeneous solution of honey). The concentrations of real samples were determinated using the standard addition method of 10 and 20 ng/mL of hIgG or 10 and 20 µM of H_2_O_2_ consecutively in order to calculate the recovery rates.

## Supporting Information

The Supporting Information features the statistical distribution of gold nanoclusters in dependance of the number of cyclic scans and supplementary electrochemical experiments (*I*–*t* transient curve, current dependence on incubation time and selectivity/specificity test of the immunosensor).

File 1 Additional experimental data.
