# Supplementary material for: Optimized design of a nanostructured SPCE-based multipurpose biosensing platform formed by ferrocene-tethered electrochemically-deposited cauliflower-shaped gold nanoparticles
Source: Beilstein J Nanotechnol. 2015 Sep 1;6:1840–52. doi: 10.3762/bjnano.6.187 (PMC4578399; doi:10.3762/bjnano.6.187)
Supplement: File 1 — Additional experimental data. [file Beilstein_J_Nanotechnol-06-1840-s001.pdf]

Supporting Information

for

Optimized design of a nanostructured SPCE-based multipurpose biosensing platform formed by ferrocene-tethered electrochemically-deposited cauliflower-shaped gold nanoparticles

Wicem Argoubi[1], Maroua Saadaoui[1], Sami Ben Aoun*[2‡] and Noureddine Raouafi*[1§]

Address: [1]University of Tunis El-Manar, Chemistry Department, Laboratory of Analytical Chemistry and Electrochemistry (LR99ES15), campus universitaire de Tunis El-Manar 2092, Tunis, Tunisia and [2]Department of Chemistry, Faculty of Science, Taibah University, PO. Box 30002 Al-Madinah Al-Munawarah, Saudi Arabia

Email: Noureddine Raouafi* - n.raouafi@fst.rnu.tn;
Sami Ben Aoun* - sbenaoun@taibahu.edu.sa

*Corresponding author

[‡]Tel.: +966590900727; Fax.: +966148628023 (Ext. 4326)
[§]Tel.: +21671872600 (Ext. 273); Fax.: +21671883424

Additional experimental data

Table S1: Statistical distribution of formed gold nanoparticles per SEM frame counted for 5 SEM frames and the density of nanoparticles per square micrometer as function of the number of cycles used for the electrodeposition.

Number of cycles	5	10	15	20
**Mean number of particles ± dev. /SEM frame**	45 ± 8	43 ± 11	64 ± 21	26 ± 15
**Density of gold nanoparticles (nanoparticles/μm^2)**	5.4 ± 0.9	5.2 ± 1.4	7.7 ± 2.5	3.2 ± 1.8

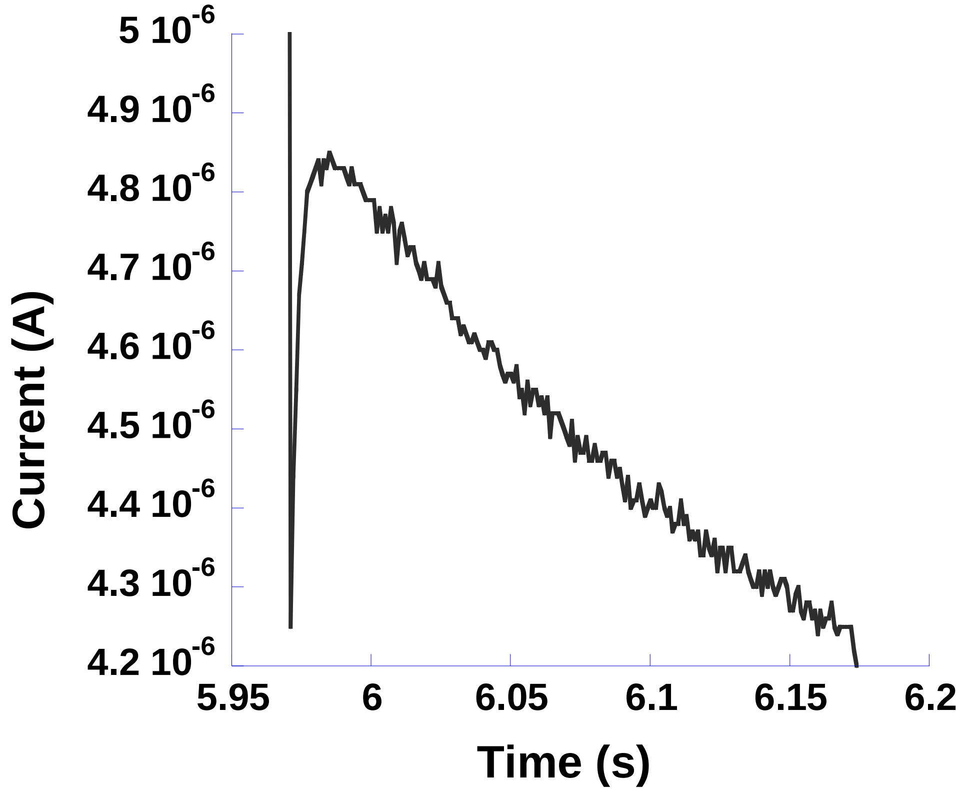

Figure S1: Current-time transients recorded for Au electrodeposition on a screen-printed carbon graphite electrode at potential of +350 mV.

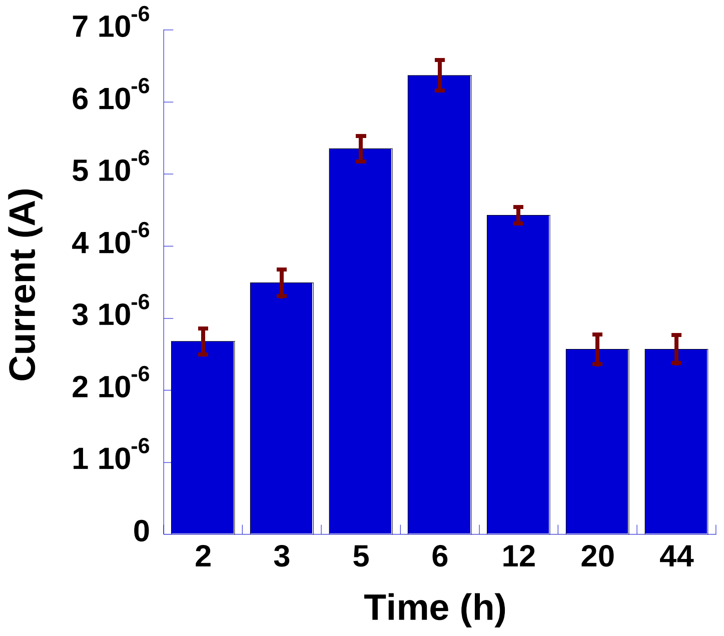

Figure S2: Optimization of the incubation time of the electrode modified in 5 mM of ferrocene derivative solution.

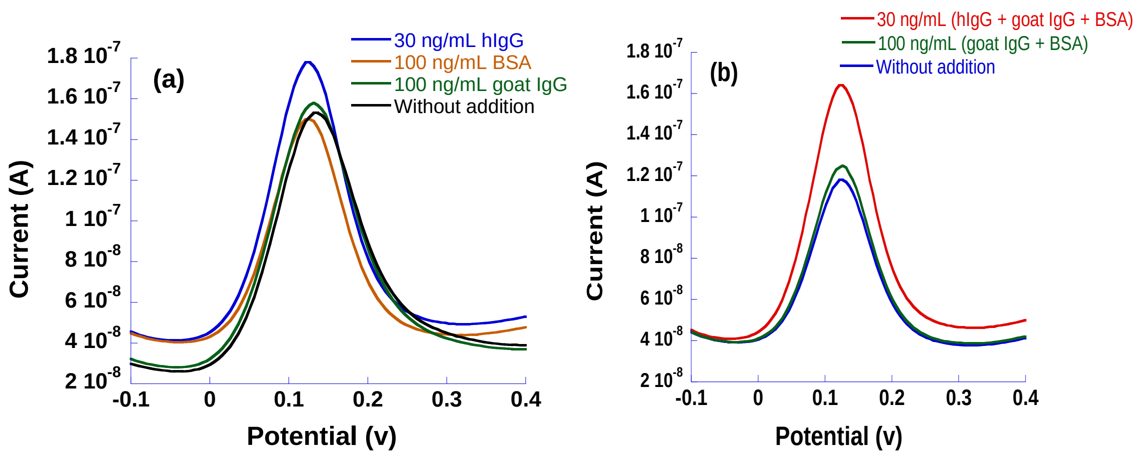

Figure S3: (a) Selectivity and (b) specificity DPV studies of the immunosensor response, immunoresponse of the hIgG sensor to BSA and gIgG interfering proteins.

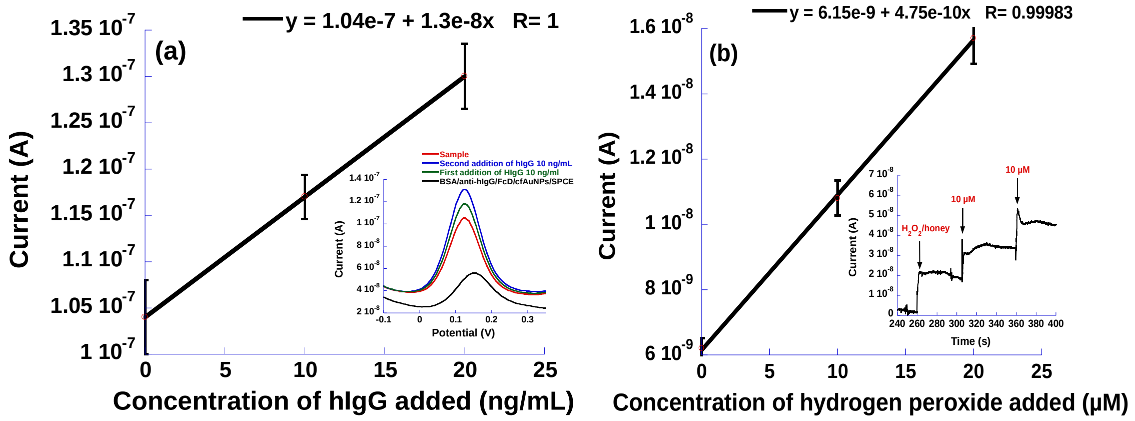

Figure S4: Determination of the concentrations of (a) hIgG and (b) H_2O_2 in real samples of serum and honey using standard addition method.
